# Association of Living Arrangement Conditions and Socioeconomic Differentials with Anemia Status among Women in Rural Bangladesh

**DOI:** 10.1155/2016/4571686

**Published:** 2016-07-19

**Authors:** Ghose Bishwajit, Sanni Yaya, Shangfeng Tang, Akmal Hossain, Yang Fan, Mahmuda Akter, Zhanchun Feng

**Affiliations:** ^1^School of Medicine and Health Management, Center for Health Research Service in Rural Areas, Key Research Institute of Humanities & Social Sciences at Universities in Hubei Province, Tongji Medical College, Huazhong University of Science and Technology, Wuhan, Hubei 430030, China; ^2^Faculty of Health Sciences, University of Ottawa, Ottawa, ON, Canada K1N 6N5; ^3^School of International Development and Global Studies, University of Ottawa, Ottawa, ON, Canada K1N 6N5; ^4^Department of Occupational & Environmental Health, Tongji Medical College, Huazhong University of Science and Technology, Wuhan, Hubei 430030, China; ^5^School of Economics and Management, Wuhan University, Wuhan, Hubei 430072, China; ^6^Department of Pharmacology, State University of Bangladesh, Dhaka 1000, Bangladesh

## Abstract

In Bangladesh, iron deficiency is the most common cause of anemia and remains a significant public health concern. Being a high anemia prevalent country, numerous efforts have been made to confront the issue especially among women and children by both local and international actors. Though the situation has substantially improved in recent years, a staggering number of adult women are currently living with anemia. The etiology of anemia is a multifactorial problem and has been proposed to be associated with various household, societal, economic, cultural factors apart from dietary habits. However, evidence regarding the household arrangements and socioeconomic determinants of anemia is scarce, especially in the context of Bangladesh. To this end, we utilized the 2011 demographic and health survey data to explore the association between anemia status and selected demographic, socioeconomic, and household characteristics. Our result showed significant correlation of anemia with both sociodemographic and household characteristics. Among the sociodemographic variables the following were found to be significantly associated with anemia status: age (*p* = 0.014; OR = 1.195; 95% CI = 1.036–1.378) and microcredit membership (*p* = 0.014; OR = 1.19; 95% CI = 1.037–1.386). Regarding the household arrangements, women utilizing biomass fuel for cooking (*p* < 0.019; OR = 1.82; 95% CI = 0.981–2.460) were more likely to be anemic.

## 1. Introduction

Despite major advances in nutritional and food sciences, micronutrient deficiencies remain a major challenge for global health. Iron deficiency anemia is one of the most prevalent nutritional disorders and thus an important contributor to global disease burden [1]. Anemia is the most commonly encountered micronutrient deficiency in the industrialized countries [2], though more debilitating in the LMICs (low- and middle-income countries). Anemia affects about a quarter and over half of the maternity related cases, respectively, in developed and developing countries [3]. There has been a growing recognition regarding the devastating consequences of IDA on public health and socioeconomic progress of nations. Besides developing increased vulnerability to morbidity and mortality from other diseases, anemia has been shown to be responsible for lower cognitive functioning and academic and professional underperformance, thereby leading to poor workforce productivity and undermining national development efforts [3, 4]. According to the estimates of a China based study, productivity gain through reduction of IDA prevalence by 30% would worth ¥107 billion (nearly $17 billion) in a decade [4]. The risks and consequences of IDA are more pronounced on women's general and reproductive health outcomes as being commonly associated with gestational and obstetric complications [5], preterm birth, low-birthweight (LBW), and perinatal and neonatal mortality [6]. As a high anemia prevalent country, these complications are also considered serious health concerns in Bangladesh. From the perspectives of the widespread undernutrition and maternal and child mortality, anemia intervention is a matter of national priority for maternal health promotion and socioeconomic empowerment of women in Bangladesh.

South Asian countries, notably India and Bangladesh, share a significant burden anemia and associated health risks [3, 5, 6]. Given the high rate of infectious diseases, worm infestations, and undernourishment, micronutrient deficiency diseases including anemia remain a major barrier for health promotion in Bangladesh [7–9]. Poor national nutrition policy coupled with household food insecurity, poor dietary diversity, low BMI (body mass index), and high parity is among the major causes of anemia among women in Bangladesh [10]. Evidence suggests that about one-third of Bangladeshi women are undernourished (BMI < 18.5 kg/m^2^) [11] and two- to three-fifth are living with mild to moderate anemia [12–14]. Though past researches focused mainly on physiological and dietary factors, a closer inspection on the underlying social determinants of health (SDH) reveals the functioning of a distal, but inextricable nexus to the problem [15].

During the past two decades, there has been a growing recognition of the fact that medical care alone is not adequate to address the emerging issues in healthcare and that a greater emphasis is required on the SDH [16]. In general terms, SDH refers to the elements of the social environment in which people were born, grow, live, work, and age [16], which influence the degree of exposure and susceptibility to the risk factors, for example, lifestyle factors, nutritional status, type of housing, and living environment. Addressing the various social and household determinants has a prominent role to play in alleviating the root causes of hidden hunger or micronutrient deficiency disorders that affect nearly two billion people worldwide [3]. Therefore, understanding and indexing these factors can be regarded as a key step towards developing appropriate policy measures. However, population based studies in the context of the SDH on anemia is scarce, especially in Bangladesh. To this end, in this study we included the pertinent socioeconomic and demographic differentials and household factors to health to observe if anemia status among Bangladeshi women varies in relation to these variables.

## 2. Methodology

Data for this study were sourced from the sixth round (latest) of Bangladesh Demographic and Health Survey (BDHS). Dataset was collected through registration in the DHS Program website. The DHS survey is nationally representative and cross-sectional in nature and was carried out in 2011 from July 8 through December 27. The survey was conducted by the National Institute of Population Research and Training (NIPORT). The survey is a part of the International Demographic and Health Survey program known as MEASURE DHS, which is currently active in 90 countries and conducted under the auspices of the United State Agency for International Development (USAID) and technical assistance of ICF International of Calverton based in USA. BDHS 2011 covered in total 17.141 households.

### 2.1. Dependent Variable

Anemia status was the dependent variable and was dichotomized as being anemic and nonanemic. We followed WHO guidelines for measuring anemia status. However, stratification of anemia was not possible due to negligible prevalence of severe anemia (Hb < 7 g/dL) among the participants. Based on hemoglobin concentration (g/dL), participants were categorized as nonanemic (Hb ≥ 11 g/dL) and anemic (Hb < 11 g/dL). For adult participants, DHS Program employs HemoCue blood hemoglobin testing system through finger prick. HemoCue is a point-of-care testing (POCT) system, easy to use, highly reliable, and one of the most commonly utilized hemoglobin testing devices in the world.

### 2.2. Covariates (Socioeconomic and Demographic) Were Categorized in the Following Way

Covariates were categorized as follows: age: 15 to 29 years/30 to 49 years; type of residency: rural/urban; educational attainment: nil (no formal education), primary (1–5 years of formal schooling), and secondary or higher (>5 years of formal schooling); wealth status: {lowest (below average)}, {middle (average)}, and {highest (above average)}; food security level: secure/insecure [HFIAS score (DHS utilizes a retrospective method called Household Food Insecurity Access Scale (HFIAS) to measure the prevalence and degree of household food insecurity. This scaling method was originally developed by USAID funded Food and Nutrition Technical Assistance (FANTA) project. The scoring system is based on responses to following yes/no questions. (1) Had three square meals in the past 12 months. (2) Skipped entire meals in the past 12 months. (3) Ate less food in the past 12 months. (4) Ate wheat or rice in the past 12 months. (5) Asked food from relatives or neighbors in the past 12 months. Score ranges from 0 to 15 with 0 representing the most secure and 15 the least secure): 0–5 = secure; 6 to 15 = insecure]; employment status (yes/no); microcredit borrower (tes/no) (based on membership with any of the following institutions: (1) Association for Social Advancement aka ASA, (2) Bangladesh Rural Advancement Committee aka BRAC, (3) Bangladesh Rural Development Board aka BRDB, and (4) Grameen Bank; number of household members: <5 members/≥5 members).

### 2.3. Living Arrangement Conditions Were Dichotomized in the Following Way

Living arrangement conditions were dichotomized as follows: water source: hygienic (tube-well, borehole, protected dug-well, and bottled water) and unhygienic (river, pond, and unprotected well); cooking fuel: clean (electricity, LPG, and natural gas) and unclean (wood, charcoal, kerosene, straw, and animal dung); toilet facility: sanitary (ventilated improved pit latrine (VIP), flush to piped sewer system, and pit latrine with slab), and insanitary (flush nonpit, pit latrine without slab/open pit, and no facility); main floor material: concrete (cement, parquet, and polished wood)/raw (earth, sand, palm, and bamboo); main wall material: concrete (cement and bricks)/raw (wood planks/shingles, bamboo with mud, and stone with mud); main roof material: concrete (roofing shingles, cement, and tiles)/raw (thatched/palm leaf, palm, and bamboo).

### 2.4. Data Analysis

Sociodemographic characteristics were tabled using descriptive statistics. Cross tabulation and *χ* ^2^ test were performed for associated probabilities and to check for statistical association between anemic and nonanemic groups across the covariates. All the covariates were used as categorical variables. Given the binary nature of the dependent variable (anemic or nonanemic), we used binary logistic regression analysis to adjust for potential confounders and examine the independent effect of the explanatory variables on anemia status. Variables which were found to have significant association from *χ* ^2^ test in cross tabulation were entered into regression model. Odds ratios were calculated to assess the likelihood of being anemic between two groups in relation to the explanatory variables. Results of regression analysis were presented as *p* values and odds ratios. A two-tailed *p* value < 0.05 was considered statistically significant for all associations. Data analyses were performed utilizing SPSS version 20 for Mac (SPSS Inc., Chicago, IL, USA).

### 2.5. Ethical Clearance

The DHS Program maintains strict standards for data confidentiality and protecting the privacy of all participants. Informed consent was obtained from all participants before each interview. In addition, the ICF International ensures that the survey complies with the US Department of Health and Human Services regulations for the protection of human subjects, and the host country ensures that the survey complies with laws and norms of the nation. Approval for this study was not required since the data is secondary and is available in the public domain. More details regarding DHS data and ethical standards are available at http://goo.gl/ny8T6X.

## 3. Results

### 3.1. Baseline Characteristics of the Study Population

The basic socioeconomic and demographic characteristics of the study population were presented in Table 1. It shows that majority of the participants were aged between 30 and 49 years. More than three-fifth of the women had completed primary education. Proportion of women passing secondary/higher secondary school was quite low (4.2%), and about one-third of the women had no formal education. One-third of the study population are from households with lower than average wealth status. Two-fifth were from middle wealth status households and only a quarter had higher than average wealth status. Regarding occupation and income status, only about 10% of the participant had a permanent employment, about 30% were microcredit borrowers. Four-fifth of the women reported living in food secure conditions (79.9%). Results regarding household arrangement shows that almost all participants reported having hygienic water sources and sanitary latrine facilities. However, rate of utilization of clean cooking fuel was quite low as only 8.7% used clean cooking fuel. Percentage of households with concrete floor, wall, and roof was 12.2, 41.4, and 87.8, respectively.

Table 1 also shows the results of cross tabulation showing the contrast between the two groups based on their anemia status and its association with the explanatory variables. Irrespective of the degree of severity, in total 1629 women were found to be living with anemia with a prevalence rate of 44.15%. Results of chi-square tests showed that anemia status was significantly associated (*p* < 0.05) with several demographic (age, total number of births, and body mass index), socioeconomic (educational attainment and microcredit), and households characteristics (cooking fuel, toilet facility, and main floor material).

Variables that proved significant correlation in the chi-square tests were entered in the regression model to measure the degree of association with anemia status.  Table 2 presents the factors of significant association between anemia and selected socioeconomic and household characteristics among the participants. The results revealed a significant association with participants age (*p* = 0.014; OR = 1.195; 95% CI = 1.036–1.378) and microcredit membership (*p* = 0.014; OR = 1.199; 95% CI = 1.037–1.386).

### 3.2. Association between Selected Household Factors and Anemia

Regarding the household characteristics, having no access to clean cooking fuel (*p* = 0.019; 95% CI = 0.981–2.460) was associated with anemia status. Though access to clean water, sanitary toilet facility, and type of main floor material showed signification association with anemia status in the chi-square tests, the association was lost in the logistic regression analysis. Odds of being anemic were 1.82-fold among women who did not enjoy clean fuel facilities.

## 4. Discussion

Anemia, like most other micronutrient deficiency disorders, is a multifactorial problem. Apart from the physiological and nutritional etiology, the occurrence of anemia can be attributed to the complex interplay of sociocultural factors, demographic and economic circumstances, education and health behaviour, healthcare organization, and social relationships [17]. Insights from previous studies suggest that a multidisciplinary and cross-cutting approach is essential to effectively address the causes that underlie the issue of anemia and other micronutrient deficiency disorders [16]. As a resource poor and highly impoverished country, Bangladesh faces extraordinary challenges in providing proper nutrition and basic living amenities for the burgeoning population. Lack of political will and research infrastructure constitutes a major obstacle to ameliorate the health and economic status of the marginalized population. However, appropriate utilization of existing resources and knowledge could generate substantial results. In Bangladesh, addressing the long-standing public health challenges like anemia will require development of a comprehensive framework based on the contextual knowledge and evidences regarding the problematic. The present study recognized a paucity of evidences regarding the social determinants of anemia in Bangladesh. In order to further validate the findings of our study, more in-depth surveys need to be conducted considering greater number of contextually relevant social and household factors.

Our findings suggest that women ageing above 30 years are more likely to be anemic compared to their younger counterparts. This can be explained by the fact that women of reproductive age has a higher requirement for iron intake compared to their male counterparts. This situation places women at a physiological disadvantage of being more susceptible to developing anemia if iron/other micronutrient intakes are chronically lower than the daily-recommended allowance.

As expected, women who had microcredit membership were less likely to be anemic. Besides contributing to economic access to better food, nutrition, and living conditions, microcredit membership can reflect an increased social involvement and exposure to health related programs and promote nutritional knowledge and status among women [18]. Microfinance organizations in Bangladesh are involved in the provision of basic essential healthcare services and health education programs and overall have a wider service coverage throughout the country [3]. Integrating anemia prevention programs with microfinance services for both members and nonmember might prove beneficial for national anemia prevention goals.

Regarding the household arrangement characteristics, type of cooking fuel showed significant association with anemia status. Though access to potable water, sanitary toilet facility, and type of main floor material were found to be correlated in the chi-square test, the association was insignificant after adjusting for the covariates. Consistent with previous findings on adult women [3], our study found that women who use biomass fuel for cooking have a higher likelihood of being anemic. In Bangladesh, especially in the rural areas, a vast majority of the households (~90%) rely on BMF as a primary source of domestic energy [19]. The potential link between biomass smoke and anemia lies in its capacity to induce systemic inflammation [20] due to its carbon monoxide and transitional metals content [1]. An Indian study reported that elevated serum levels of these compounds were found among women with chronic exposure to smoke of biomass fuel [20]. According to previous studies, more than 70% of the population in Bangladesh lack connection electricity grid [21]. Rural areas of Bangladesh lack proper supply of natural gas and electricity. As a consequence households have to rely on tress branches and leaves as cheap sources of fuel. Provision of electricity and other clean energy sources may induce women to switch from biomass fuel to electronic heater or other nonharmful cooking devices and may contribute to reduced incidence of anemia [19].

## 5. Conclusion

We conclude that besides the sociodemographic differentials, household living arrangement also has certain contributions to the epidemiology of anemia which requires consideration in designing anemia prevention and intervention frameworks. A better cooperation between sociologists and public health professionals can play a pivotal role in exploring the most pressing needs and to meet them in a sustainable manner. As one of the most malnourished nations, policy makers should attempt to formulate nutrition sensitive agriculture and health policies and integrate with a broader national development agenda for long-term success.

## 6. Strengths, Limitations, and Future Research Directions

To the best of our knowledge, this is the first population based study to explore the combined effect of demographic, socioeconomic, and living arrangement characteristics on anemia status in Bangladesh. Study sample was relatively large and included a broad set of explanatory variables. Despite all these, few important limitations have to be noted. The survey was conducted about four years back and the prevalence of the variables may have changed. Data being secondary, we did not have control over the selection and measurement of the variables (e.g., anthropometric and biochemical tests). We also did not stratify anemia level due to low nonanemia to severe and mild anemia ratio. BMF included several types (wood, charcoal, kerosene, straw, and animal dung) each of which may have different mechanism and impact on anemia. Given the high rate of BMF use, future researches should focus on measuring the individual impact of different BMF.

## Figures and Tables

**Table 1:** Baseline characteristics of the study population.

Variable	*N* (%)	Anemia status		*χ* ^2^	*p* value
		Yes	No		
		(*n* = 1629, 44.15%)	(*n* = 2065, 55.9%)		
*Age*				6.229	0.007^*∗*^
15–29	(1777) 48.1	45.8	49.9		
30–49	(1917) 51.9	54.2	50.1		
*Educational attainment *				8.81	<0.017^*∗*^
Nil	(1103) 29.9	32.0	28.2		
Primary	(2436) 65.9	64.5	67.1		
Secondary/higher	(155) 4.2	3.6	4.7		
*Wealth*				3.99	0.136
Lowest	(1236) 33.5	32.4	34.3		
Middle	(1520) 41.1	40.7	41.5		
Highest	(938) 25.4	26.9	24.2		
*Employed*				1.153	**0.115**
Yes	(393) 10.6	8.1	9.1		
No	(3301) 89.4	91.9	90.9		
*Food security level*				0.148	0.710
Secure	(2951) 79.9	80.2	79.7		
Insecure	(743) 20.1	19.8	20.3		
*Microcredit*				7.442	0.004^*∗*^
Yes	(1074) 29.1	68.6	72.7		
No	(2620) 70.9	31.4	27.3		
*Number of household members *				0.630	0.224
>5 members	(2207) 59.7	39.5	40.8		
≥5 members	(1487) 40.3	60.5	59.2		
*Water*				0.805	0.242
Piped/hygienic	(3666) 99.2	99.4	99.1		
Others/unhygienic	(28) 0.8	0.6	0.9		
*Toilet facility *				19.424	**0.0328**
Sanitary	(3685) 99.8	31.7	29.1		
Nonsanitary	(9) 0.2	68.3	70.9		
*Cooking fuel *				3.219	0.041^*∗*^
Unclean	(3374) 91.3	88.3	90.2		
Clean	(320) 8.7	11.7	9.8		
*Main floor material*				7.630	**0.012**
Concrete	(452) 12.2	11.5	12.8		
Raw	(3242) 87.8	88.5	87.2		
*Main wall material*				0.312	0.137
Concrete	(1531) 41.4	58.1	58.9		
Raw	(2163) 58.6	41.9	41.1		
*Main roof material*				1.912	0.097
Concrete	(3242) 87.8	60.5	59.2		
Raw	(452) 12.2	39.5	40.8		

^*∗*^Significant at *p* < 0.05.

**Table 2:** Association between selected socioeconomic, demographic, and living arrangement factors and anemia among women ageing between 15 and 49 years in Bangladesh, 2011.

Variable	(AOR) 95% CI	(COR) 95% CI
*Age* (15–29)^a^		
30–49	(1.168) 1.127–1.211	(1.158) 0.992–1.351
*Educational attainment* (secondary/higher)^a^	—	
Primary	(0.723) 0.594–0.880	(0.746) 0.593–0.939
Nil	(0.805) 0.513–1.264	(0.833) 0.666–1.043
*Microcredit* (yes)^a^	—	
No	(1.175) 1.150–1.12	(1.178) 1.024–1.355
*Toilet facility* (sanitary)^a^	—	
Insanitary	(1.121) 1.096–1.147	(1.387) 0.344–5.596
*Cooking fuel* (clean)^a^	—	
Unclean (biomass fuel)^a^	(1.82) 0.981–2.460	(1.413) 1.073–1.863
*Main floor material* (concrete)^a^	—	
Raw	(1.140) 0.984–1.321	1.250 (1.166–1.339)

*Notes*. a: reference category. OR: odds ratio.

## Abbreviations

IDA: Iron deficiency anemia
LMICs: Low- and middle-income countries
LBW: Low-birthweight
SDH: Social determinants of health
BDHS: Bangladesh Demographic and Health Survey
POCT: Point-of-care testing.
